# Current Perspectives on the Role of TNF in Hematopoiesis Using Mice With Humanization of TNF/LT System

**DOI:** 10.3389/fimmu.2021.661900

**Published:** 2021-05-13

**Authors:** Violetta S. Gogoleva, Kamar-Sulu N. Atretkhany, Arina P. Dygay, Taisiya R. Yurakova, Marina S. Drutskaya, Sergei A. Nedospasov

**Affiliations:** ^1^ Center for Precision Genome Editing and Genetic Technologies for Biomedicine, Engelhardt Institute of Molecular Biology, Russian Academy of Sciences, Moscow, Russia; ^2^ Department of Immunobiology and Biomedicine, Sirius University of Science and Technology, Sirius, Russia; ^3^ Engelhardt Institute of Molecular Biology, Russian Academy of Sciences, Moscow, Russia; ^4^ Department of Immunology, Faculty of Biology, Lomonosov Moscow State University, Moscow, Russia

**Keywords:** cytokines, cytokine blockade, steady-state hematopoiesis, emergency hematopoiesis, humanized mouse models

## Abstract

TNF is a multifunctional cytokine with its key functions attributed to inflammation, secondary lymphoid tissue organogenesis and immune regulation. However, it is also a physiological regulator of hematopoiesis and is involved in development and homeostatic maintenance of various organs and tissues. Somewhat unexpectedly, the most important practical application of TNF biology in medicine is anti-TNF therapy in several autoimmune diseases. With increased number of patients undergoing treatment with TNF inhibitors and concerns regarding possible adverse effects of systemic cytokine blockade, the interest in using humanized mouse models to study the efficacy and safety of TNF-targeting biologics *in vivo* is justified. This Perspective discusses the main functions of TNF and its two receptors, TNFR1 and TNFR2, in steady state, as well as in emergency hematopoiesis. It also provides a comparative overview of existing mouse lines with humanization of TNF/TNFR system. These genetically engineered mice allow us to study TNF signaling cascades in the hematopoietic compartment in the context of various experimental disease models and for evaluating the effects of various human TNF inhibitors on hematopoiesis and other physiological processes.

## Introduction to Hematopoiesis

Hematopoiesis is the process of blood cell development that in vertebrates is initiated early during embryogenesis and may be divided into 3 phases or so-called distinct waves of hematopoiesis.

**The first** (or **primitive**) wave takes place in the yolk sac starting in mice at embryonic day 7.5 (E7.5) and generates unipotent blood cell types (1). **The second** (or **pro-definitive**) wave occurs in the yolk sac, embryo proper and allantois of the mouse embryo and gives rise to multipotent progenitors (2). **The third wave** of hematopoiesis represents **definitive** hematopoiesis and is dependent on the activity of hematopoietic stem cells (HSCs), which are the basic units of the adult hematopoietic system. HSCs generated in the embryonic aorta-gonad-mesonephros region first colonize the fetal liver (E10.5) and then shortly before birth (E16) migrate to the bone marrow (BM), where the majority of HSCs reside to sustain steady state hematopoiesis (3).

HSCs are multipotent, self-renewing cells capable of differentiating into all mature blood cell lineages over the lifespan of the animal. Lineage choice may be directed both intrinsically and extrinsically *via* activation of transcription factors or extrinsically by cytokines (4). The majority of HSCs are quiescent under steady-state conditions, and few HSCs cycle to sustain hematopoiesis (5). In order to maintain hematopoietic homeostasis and to prevent development of malignancies, the self-renewal and differentiation capacities of HSCs are tightly regulated. This is, at least partly, achieved by the specialized network of interactions between distinct cell types (6, 7) and secreted factors (8, 9) in the BM niche that maintains HSC activity in steady-state conditions.

However, in the case of systemic infections and pathological conditions, such as myeloablation after chemo- or radiotherapy, some HSCs may respond and exit their quiescent state. These ‘activated’ HSCs contribute to the pool of hematopoietic progenitor cells, which will undergo further differentiation in order to replenish the population of immune cells being in high demand at the sites of inflammation in the process of so-called **emergency hematopoiesis**. This is possible because hematopoietic stem and progenitor cells (HSPCs) express Toll-like receptors (10) and cytokine receptors (11) and, thus, can respond to inflammatory signals. Activation of TLR signaling in HSPCs not only drives myeloid cell differentiation (10), but also leads to production of cytokines, which regulate myeloid differentiation and HSPC proliferation (12). HSPCs may respond to cytokines released during inflammation either systemically or locally by cells in the hematopoietic microenvironment or BM niche. Indeed, it was shown that HSPCs express various cytokine receptors, including IL-1R (13), IL-6Rα, as well as both TNF receptors, TNFR1 and TNFR2 (12).

As mentioned above, proinflammatory cytokines are critical components of inflammation-induced myelopoiesis. However, inflammatory signals may also be implicated in the maintenance of homeostatic hematopoiesis. During embryonic development proinflammatory cytokines control HSPC specification in the pro-definitive wave of hematopoiesis (14). Moreover, proinflammatory cytokines may regulate adult hematopoiesis and maintain the balance between HSC dormancy and lineage commitment (15). This question is important because systemic and long-term anti-cytokine therapy is being applied to treat an increasing number of conditions, including autoimmune disorders. One of the proinflammatory cytokines implicated in hematopoiesis is TNF, which we discuss in the context of humanized mouse models in this Perspective.

## TNF in Steady-State Hematopoiesis

**Figure 1:**
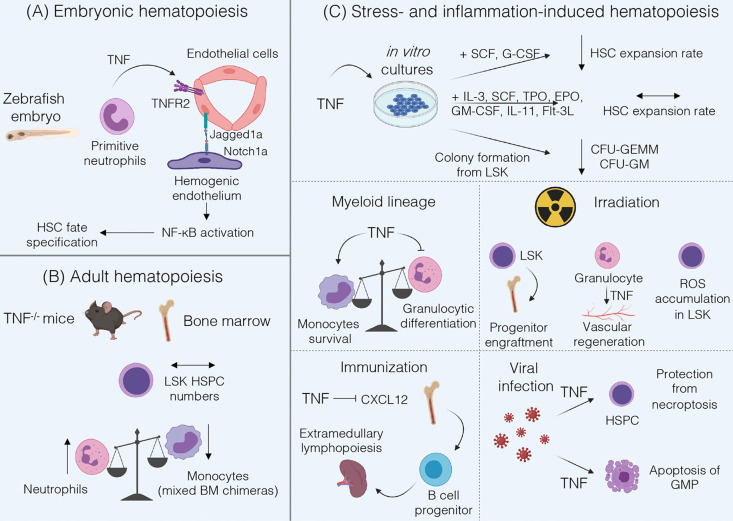
Summary of TNF functions in hematopoiesis. **(A)** During fetal hematopoiesis in zebrafish TNF/TNFR2 signaling is required to establish HSC fate *via* activation of Notch and NF-kB signaling (18). **(B)** Bone marrow of adult TNF-deficient mice is characterized by normal LSK HSPC numbers and by an increase in Gr-1^+^ neutrophils (19). Mixed *Tnf* ^-/-^ and *Tnf* ^+/+^ BM chimeras underrepresent TNF-deficient monocytes (20). **(C)** TNF may inhibit HSC expansion when cultured with SCF and G-CSF, but not in cytokine-rich medium (21). Addition of TNF to LSK cultures inhibits formation of CFU-GEMM and CFU-GM (22). TNF promotes monocytes survival (20) and inhibits proliferation and differentiation of granulocyte progenitors (19, 23, 24). Under inflammatory conditions induced by irradiation TNF may be beneficial for progenitor engraftment (25) but stromal cell-derived TNF induces ROS accumulation in HSPCs (26), and granulocyte-derived TNF is involved in vascular regeneration (27). Following immunization, TNF may suppress CXCL12-dependent retention of B cell progenitors in the bone marrow leading to their migration (28). In the case of viral infections TNF protects HSPCs from necroptosis, enhances myelopoiesis and induces apoptosis of GMP (21).

TNF is a pleiotropic cytokine involved in inflammation, development of secondary lymphoid organs and immune regulation. TNF is produced as a transmembrane protein and can be proteolytically cleaved into a soluble form. TNF exerts its functions *via* two distinct receptors – TNFR1 and TNFR2 (16). Both receptors also may interact with soluble (LTα_3_) and membrane-bound (LTα_2_β_1_) lymphotoxins, respectively (17). Interestingly, TNF may play a role both in embryonic and in adult hematopoiesis (**Figure 1**).

The role of TNF during fetal hematopoiesis was mostly studied in zebrafish. These studies revealed that TNF derived from primitive neutrophils binds to TNFR2 on endothelial cells resulting in the upregulation of Notch ligand Jagged1a, which in turn binds to Notch1a receptor on the neighboring hemogenic endothelium triggering HSC fate specification. Moreover, TNF/TNFR2 axis also activates canonical NF-κB pathway in hemogenic endothelium, which triggers a transcriptional program to establish HSC generation (18). Of note, not only TNF but also other inflammatory stimuli such as TLR4-MyD88 signaling or G-CSF may lead to NF-κB activation required for HSC development (29). Interestingly, inflammatory signaling represents a highly conserved pathway regulating the HSC development. Studies in E9.5 mouse embryos revealed that hematopoietic cluster cells and endothelial cells respond to IFNγ and to a lesser extent to TNF stimulation. The most likely source of TNF in the mouse embryo is the population of primitive F4/80^+^ macrophages, similar to the situation in zebrafish embryo (30). However, the precise contribution of TNF to mammalian HSC development is not completely understood. Taken together, TNF signaling may be required for HSC emergence in the developing embryo *via* activation of evolutionarily conserved signaling pathways, but it might be partially redundant with other inflammatory stimuli.

Many studies have been performed to examine the role of TNF in the adult hematopoiesis; however, most of these have relied on cell culture and/or bone marrow chimeras, which could indirectly affect HSPC phenotype and functions. Another challenge in understanding the role of TNF in steady-state conditions is due to its capacity to induce systemic inflammation when administered *in vivo* which in turn may activate stress-induced hematopoiesis thus obscuring TNF contribution to hematopoiesis. Therefore, in this section we will focus on *in vivo* studies using gene-deficient mice. TNF deficiency did not alter the number of Lin^-^Sca-1^+^c-kit^+^ (LSK) in the BM, consisting mostly of lineage-biased multipotent progenitors (19). In early studies TNFR1-deficient bone marrow was characterized by increased number of LSK (22). However, examination of purified LSKFLT3^-^ HSPCs revealed no differences in the numbers of HSPC isolated from the adult BM of TNFR1/TNFR2 double knockout mice (31). Hence, it is likely that TNF does not affect HSPC compartment under steady-state conditions *in vivo*.

Regarding the differentiation of HSCs to more committed progenitors, TNFR1-deficient mice showed a decrease in pre-B cell compartment and an increase in myeloid progenitors (32). Accordingly, TNF-deficient mice demonstrated an increase in the number of Gr-1^+^ neutrophils both in the BM and in peripheral blood (19). Transcriptome analysis of monocytes and their BM precursors revealed an increase in TNF expression upon differentiation of Ly6C^hi^/Ly6C^int^ monocytes into Ly6C^lo^ monocytes (20). Therefore, TNF may control granulocyte number in the blood and BM and support monocytic differentiation *in vivo*.

## TNF in Stress- and Inflammation-Induced Hematopoiesis

Numerous studies on the role of TNF in HSPC functions relied on *in vitro* and *in vivo* colony formation assays together with the assessment of reconstitution potential, engraftment and survival abilities of multipotent progenitors upon transplantation into irradiated recipient mice. However, results obtained from these studies should be carefully interpreted since these setups may affect HSPC proliferation, survival, self-renewal and differentiation. We will discuss some experiments and their possible applicability to hematopoietic compartment.

Studies with competitive co-transplantation of TNFR1^-/-^TNFR2^-/-^ CD45.2^+^ and wild-type (WT) CD45.1^+^ BM cells into lethally irradiated congenic CD45.1^+^CD45.2^+^ WT recipients showed enhanced activity of TNFR1^-/-^TNFR2^-/-^ HSCs as determined by long-term reconstitution by TNFR-deficient HSCs following transplantation. Moreover, *in vivo* administration of TNF to WT mice led to a decrease in BM cellularity and to reduction in HSC cycling activity in a competitive transplantation assay (31). These data suggest that TNF blockade may be beneficial for post-transplantation reconstitution and also supports the idea that TNF may suppress HSC activity. In contrast, transplantation of bone marrow cells from 6 months old TNFR1-deficient mice into lethally irradiated recipients showed reduced repopulating ability of TNFR1^-/-^ BM cells as compared to WT cells (32). However, this effect was shown on non-purified HSPCs and under long-term transplantation conditions that may affect the outcome of the experiment. TNF does not inhibit expansion of highly purified Lin^-^Sca-1^+^c-kit^+^Flk2^-^CD150^+^CD48^-^ HSCs in medium supplemented with IL-3, SCF, TPO, EPO, GM-CSF, IL-11 and Flt3-L, or so-called cytokine-rich medium, regardless of TNF concentration. Under cytokine-poor conditions (medium supplemented with SCF and G-CSF only) HSC expansion rate upon addition of TNF was significantly decreased (21). Similar findings were reported by Pronk et al. when TNF was added to the culture of LSK cells (31). Importantly, studies by Yamashita et al. revealed that this effect was due to the inhibition of autophagy by TNF, which sensitized HSCs to cell death in cytokine-deprived environment (21). Taken together, HSCs appear to be resistant to TNF cytotoxicity but this resistance may be changed by the environmental stress.

Addition of exogenous TNF to bone marrow cultures of LSK resulted in reduced numbers of large-sized colonies, such as CFU-GEMMs (colony-forming unit − granulocyte, erythroid, macrophage, megakaryocyte) and CFU-GMs (granulocyte/macrophage colony forming units), in methylcellulose assay (22). LSK isolated from TNF-deficient mice gave rise to an increased number of splenic colony-forming units in lethally irradiated recipient mice (19). Experiments with TNFR1 and TNFR2 agonists demonstrated that TNFR2 is essential for TNF-mediated inhibition of colony formation of early Lin^-^Sca-1^+^ progenitors, while addition of TNFR1 agonist had no significant effect on the number of Lin^-^Sca-1^+^ proliferative clones (23). Thus, TNF may inhibit formation of CFU-GEMM and CFU-GM colonies and differentiation potential of multipotent progenitors, presumably *via* TNFR2.

Evaluation of TNF effects on myeloid lineage commitment revealed that addition of TNF or TNFR1 agonists resulted in a decrease in G-CSF-induced colony formation and in inhibition of G-CSF expression by BM cultures (23). This was further supported *in vitro* in TNF-deficient long-term bone marrow cultures, which were characterized by increased proliferative potential of granulocytic progenitors and increased numbers of CFU-GMs within sorted LSK population, as compared to WT BM cultures (19). Consistent with *in vitro* data, CFU-GM formation was reduced in WT-recipient mice reconstituted with TNF-deficient BM, suggesting a possible role of TNF, expressed by hematopoietic and not by stromal cells, in inducing cell death at the GMP stage (33). This is also in agreement with data showing elimination of GMPs by TNF in a dose-dependent manner (21). Next, TNF was demonstrated to block granulocytic differentiation and IL-3-dependent proliferation of granulocyte-committed cells (24). At the same time, an autonomous effect of TNF on monocytes was proposed. Specifically, TNF is required for monocyte survival *in vivo* (20). Thus, TNF stands at the crossroad of myeloid lineage commitment *via* negatively affecting granulocytic cell differentiation and driving the survival of monocytes. Interestingly, these findings suggest possibilities to control the differentiation of myeloid progenitors. For example, TNF was shown to directly upregulate a central transcription factor for myeloid lineage commitment, PU.1, in HSPC *in vivo* during acute inflammation (34). Moreover, in some abnormal hematopoiesis conditions, such as clonal hematopoiesis associated with aging, TNF blockade may help to overcome TET2-mutant HSPC skewing toward myeloid lineage and the formation of CFU-GMs (35).

Other studies revealed that addition of TNF to the LSK cultures negatively regulated both long-term and short-term reconstituting activity after transplantation of LSK into lethally irradiated recipient mice (36). However, TNF produced by BM microenvironment is required for long-term engraftment and survival of purified LSK in allogeneic and syngeneic recipients (37). This is in line with the fact that the engraftment of Lin^-^ BM cells from TNFR1- and TNFRs-deficient donors in wild-type recipients was defective suggesting a stimulatory role of TNF in successful progenitor engraftment. Interestingly, homing of the engrafted progenitors to the BM was primarily mediated by TNFR1 (25). On the other hand, total body irradiation and inflammation within BM was associated with elevated levels of TNF. Subsequently, TNF induced ROS accumulation in LSK leading to impairment of their reconstitution ability. Addition of TNFR1 antagonistic peptide to LSK cultures inhibited ROS accumulation suggesting that TNFR1 blockade prior to transplantation may lead to improved reconstitution capability (26). Altogether, more precise *in vivo* studies with defined protocols of total body irradiation and transplantation are needed to identify the role of TNF in the engraftment and survival of HSPCs. For example, TNF may contribute to regeneration of BM niche after HSC transplantation, since TNF-deficient mice displayed reduced number of BM endothelial cells upon myeloablation with a single injection of 5-fluorouracil. Furthermore, Gr1^+^CD115^-^ granulocyte-derived TNF promoted vascular regeneration following transplantation (27). In the context of hematological malignancies, such as myeloproliferation, inflammation may be implicated in the disruption of BM microenvironment. *Flt3* ^ITD/ITD^ mice, harboring the most common somatic mutation in patients with acute myeloid leukemia, were shown to upregulate TNF production by endothelial cells in the BM niche, which subsequently may lead to suppression of HSC activity. Treatment of *Flt3* ^+/+^ and *Flt3* ^ITD/ITD^ mice with Etanercept resulted in partial rescue of LSKCD150^+^CD48^-^ engraftment capacities (38).

As a consequence of inflammation, TNF may mobilize B cell progenitors from BM to peripheral tissues by suppressing their CXCL12-induced retention in the BM. This mobilization establishes extramedullary lymphopoiesis possibly needed for resolution of inflammation (28). Contribution of TNF to emergency myelopoiesis was clearly demonstrated by Yamashita et al. (21). In a model of poly(I:C)-induced inflammation, TNF, on one hand, induced NF-kB activity, which protected HSPCs from inflammation-induced necroptosis and, on the other hand, promoted myelopoiesis and induced apoptosis of GMPs (21). This effect may be protective in pathogenesis of some viral infections, in which case GMPs may act as the latent reservoirs for viruses in the BM and should be eliminated (39).

Defects in hematopoiesis may lead to the development of hematologic disorders. Although the exact role of TNF in different hematological diseases remains incompletely understood, elevated levels of TNF were found in patients suffering from myeloid leukemia (40) and myelodysplastic syndromes (41, 42), Fanconi anemia (43), Hodgkin’s disease (44) and Non-Hodgkin lymphoma (45).

Moreover, since TNF inhibitors are widely used to treat autoimmune disorders, it is important to address possible side effects of anti-TNF therapy. Indeed, hematological complications were reported in patients on TNF blockade (**Table 1**). For example, a case report was published showing pancytopenia after treatment with Infliximab (47). Furthermore, side effects of Etanercept [which also neutralizes both LTα_3_ and LTα_2_β_1_ (17)] treatment on hematopoietic cells have been reported including aplastic anemia (52), thrombocytopenia (55), and bone marrow aplasia with pancytopenia (64). Interestingly, a common phenomenon in patients receiving anti-TNF therapy is the development of neutropenia (81). Apart from that, some lymphoproliferative disorders were reported. For example, treatment of rheumatoid arthritis and inflammatory bowel disease (IBD) patients with Etanercept or Infliximab, respectively, led to formation of cutaneous and systemic T-cell lymphomas (75). A case of Hodgkin-type lymphoproliferative lesions was reported for an IBD patient treated with Infliximab for a 6-month period (79).

**Table 1:** Hematological complications from systemic TNF blockade.

Adverse effect		Diagnosis	Treatment	Brief report	Onset upon anti-TNF treatment	Reference
Pancytopenia		Juvenile idiopathic arthritis	Etanercept	2/61 cases	After 0,5-12 months	(46)
		Scleroderma	Infliximab	45F case Previously diagnosed with anemia, ANA positive	After 2 weeks	(47)
		RA	Infliximab + MTX (Previously leflunomide)	66M case BM hypoplasia	After 10 days	(48)
		Indeterminate colitis	Infliximab + antibiotics	32F case	After 6 days	(49)
		RA	Etanercept	1/1073 case	NR	(50)
		RA	Etanercept + MTX	68F case	After 3 weeks	(51)
Aplastic anemia		RA	Etanercept	78M case	After 16 weeks	(52)
Thrombocytopenia		Psoriatic arthritis	Infliximab	1/16 case	After 12 weeks	(53)
		Crohn’s disease	Infliximab	15M case Platelet associated autoantibodies positive	After 6 days	(54)
		RA	Etanercept	2/1073 cases	NR	(50)
		RA	Etanercept	44F case Autoantibodies negative	After 1,5 weeks	(55)
		RA	Infliximab + MTX	56F case ANA positive dsDNA autoantibodies positive	After 28 months	
		Scleroderma overlap/rheumatoid arthritis	MTX + Prednisone + Infliximab	44F case Anticardiolipin antibodies positive ANA positive Anticentromere antibodies positive	After 13 months	(56)
		Crohn’s disease	Infliximab then Adalimumab (Previously metronidazole and azathioprine)	42F case Platelet associated antibodies positive Increased BM megakaryocytes Anticardiolipin antibodies negative	30 weeks after Infliximab treatment 1 week after Adalimumab treatment	(57)
		Psoriatic arthritis	Etanercept	61M case Autoantibodies negative	After 2 months	(58)
		Psoriasis	Etanercept	1/39 case ANA positive	After 9 weeks	(59)
		Psoriatic arthritis	Infliximab	1/26 case Autoantibodies negative	After 29 weeks	
		Psoriasis	Infliximab	2/26 cases ANA positive Antiplatelet antibodies positive	After 30 weeks	
		Crohn’s disease	Infliximab	75M case	After 14 weeks	(60)
		Ulcerative colitis	Adalimumab + azathioprine + mesalazine	54F case Anemia Autoantibodies negative	After 4 years	(61)
Thrombocytopenia and leucopenia		Juvenile idiopathic arthritis	Etanercept	2/95 cases	NR	(62)
Thrombocytopenia and neutropenia		RA	MTX + Infliximab	60F case BM hypoplasia Autoantibodies negative	After 7 weeks	(63)
Bone marrow aplasia with pancytopenia		RA	Etanercept (Previously MTX + folic acid + Hydroxychloroquine; Leflunomide and Adalimumab discontinued months before Etanercept)	62F case Autoantibodies negative	After 23 days	(64)
Neutropenia		RA	Etanercept	1/208 case	NR	(53)
		RA	Etanercept	2/207 cases		
		Spondyloathropathy/Crohn’s disease	Infliximab	20M case Neutrophil-associated antibodies	After 4 weeks	(65)
		RA	Adalimumab	3/21 cases Adalimumab 13/75 cases Etanercept 3/23 cases Infliximab 7/65 cases ANA positive	After 1 week – 26 months	(66)
			Etanercept			
			Infliximab			
		RA	Adalimumab + MTX	53F case T-cell lymphocytosis (large granular lymphocytes)	After 13 months	(67)
		Sacroiliitis	Salazopyrine + MTX + Etanercept	37F case	After 6 months	(68)
		RA	Etanercept (Previously MTX)	57F case Asymptomatic neutropenia during MTX Increased BM immature granulocyte production ANA positive Neutrophil-associated antibodies negative	After 7 weeks	(69)
		Psoriatic arthritis	Etanercept (Previously MTX)	61M case Persistent leucopenia Neutropenia during MTX Normal BM hematopoiesis ANA positive Neutrophil-associated antibodies negative	NR	
		RA	Etanercept	50F case Previous asymptomatic neutropenia Normal BM hematopoiesis ANA positive Neutrophil-associated antibodies negative	After 17 days	
		RA	Adalimumab + MTX + Prednisone	55F case ANA negative dsDNA antibodies negative	After 1 month	(70)
		RA	Adalimumab 6/31 Etanercept 49/267 Infliximab 14/69	56/298 cases	NR	(71)
		Psoriatic arthritis		7/31 case		
		Ankylosing spondylitis		6/38 cases		
		RA	Etanercept + MTX	64F case	After 2 weeks	(72)
		Ankylosing spondylitis	Etanercept	36M case	After 2 months	
		RA	Etanercept then Etanercept re-challenge then Adalimumab	65M case	3 months after Etanercept onset 2^nd^ neutrophil drop after Etanercept re-challenge Modest neutrophil drop after Adalimumab treatment	
		RA	Etanercept then Etanercept re-challenge then 2^nd^ Etanercept re-challenge	71F case	6 months after Etanercept treatment 16 months after Etanercept re-challenge 8 weeks after 2^nd^ Etanercept re-challenge	
		RA	Etanercept (Previously hydroxychloroquine + prednisolone)	42F case	After 4 injections	
		75/235 Crohn’s disease 21/46 Ulcerative colitis	Adalimumab Certolizumab Infliximab Golimumab + MTX/azathioprine/5-ASA	58/157 cases Adalimumab 2/6 cases Certolizumab 36/117 cases Infliximab 0/1 Galimumab 1/5 + MTX 77/188 + azathioprine 37/65 + 5-ASA	After 1 day – 6 years Median 1 year	(73)
Neutropenia and leucopenia		Ankylosing spondylitis	Etanercept + butazolidine then Etanercept re-challenge then Infliximab	50M case	3 weeks after Etanercept treatment 3 weeks after Etanercept re-challenge After 2^nd^ Infliximab infusion	(74)
Leucopenia		RA	Etanercept	4/1073 cases	NR	(50)
Myelodysplastic syndrome		RA	Etanercept	1/1073 case	NR	(50)
Lymphoma	NR	RA	Etanercept	3/1073 cases	NR	(50)
	Cutaneous T-cell lymphoma	Psoriatic arthritis	Etanercept	69M case	Erythroderma after 18 months	(75)
	Systemic anaplastic large cell lymphoma (ALCL)	Crohn’s disease	Infliximab	81F case	After 5 months	
	Chronic myeloid leukaemia (CML)	RA	Infliximab + MTX	56F case	After 6 months	(76)
	16 non-Hodgkin’s lymphomas 1 Hodgkin’s disease 1 Type B1 thymoma	15/18 RA 2/18 Psoriatic arthritis 1/18 NS	Etanercept 9/18 +MTX 4/18 prior MTX 4/18 prior other immunosuppressive drugs	18 cases Median age 64 years 2 cases non-Hodgkin’s lymphoma recursion 1 case nodular sclerosing Hodgkin’s disease recursion	After 2-52 weeks	(77)
	5 non-Hodgkin’s lymphomas 3 Hodgkin’s lymphomas	3/8 RA 5/8 Crohn’s disease	Infliximab 2/8 +MTX 1/8 + 6‐mercaptopurine	8 cases Median age 62 years	After 2-44 weeks	
	Non-Hodgkin’s lymphomas Hodgkin’s lymphomas	RA	Infliximab	6/5233 cases	NR	(78)
		RA	Etanercept	5/2149 cases		
	Hodgkin-like lymphoproliferative disorder	Ulcerative colitis	Infliximab	74M case	After 3 months	(79)
	4 Hodgkin lymphomas 4 B-cell lymphomas 1 metastatic lymphoma	Crohn’s disease	Infliximab	9/1541 cases	NR	(80)

‘+’ treatments were taken concomitantly. ANA, anti-nuclear antibody; BM, bone marrow; MTX, methotrexate; NR, not reported; NS, not specified; RA, rheumatoid arthritis.

Altogether, TNF may play a crucial role in inflammation-induced hematopoiesis and may be implicated in the pathogenesis of some hematologic disorders. Anti-TNF therapy may lead to rare but severe side effects affecting hematopoietic compartment and resulting in the development of hematological complications and even malignancies. The exact mechanisms of these side effects are not well understood and should be addressed in the future using humanized mouse models.

## Humanized Mice as the Tools to Study Hematopoiesis and to Evaluate the Consequences of Systemic Cytokine Ablation

To identify the effects and to evaluate the efficacy and safety of clinically available or novel human TNF (hTNF) inhibitors proper animal models should be generated and validated. Importantly, in spite of a conservative nature of TNF family of cytokines and their corresponding genes, most hTNF inhibitors do not block mouse TNF (82). Therefore, various panels of humanized mice that express hTNF and/or TNFRs are required to facilitate the research (**Table 2**). Such preclinical models were first generated in 1991 by G. Kollias group, when the first mice with overexpression of human TNF were reported (83).

**Table 2:** Humanization of TNF/LT system in mice as a tool to study human hematopoiesis.

Humanized mouse line		Expression specificity	Hematopoiesis-unrelated phenotype	Hematopoiesis-related phenotype	References
hTNF Tg (Tg197)	High copy number	Systemic	Severe polyarthritis as early as 3-4 weeks after birth Spontaneous spanial disc herniation	Mild microcytic hypochromic anemia Decrease in frequency of Sca1^+^ progenitor cells and granulocytes Increased frequency of lymphoid and monocytic origin in the bone marrow	(83–85)
	Low copy number	Systemic	Progressive arthritis at a later age Reduced body weight Increased metabolic rate Restricted motor activity		(86)
ihTNFtg	Doxycycline-inducible	Systemic	Psoriatic arthritis Keratinocyte activation Joint and skin inflammation		(87)
CD2-TNF		T cell	Progressive weight loss Vascular thrombosis Tissue necrosis Lymphoid tissue abnormalities Reduced thymic cellularity Enlarged mesenteric lymph nodes almost without lymphocytes		(88)
GFAP-wtTNF		Astrocytes	Lethal neuroinflammation Ataxia, imbalances, seizures, relapsing hind limb paralysis Diminished weight gain		(89)
GFAP-tmTNF			Severe neuroinflammation Complete hind limb paralysis Normal weight gain		
NFL-wtTNF		Neurons	Severe neuroinflammation Hind leg paralysis		
NFL-tmTNF			No abnormalities		
hTNF/LT Tg		Systemic	Thymic atrophy Impaired transition of DN1 thymocytes to the DN2		(90)
hTNFR1 Tg		Systemic	Prophylactic administration of TNFR1 antagonist leads to EAE amelioration and delayed disease onset		(91)
hTNFKI		Systemic	Pharmacological TNF inhibition with Etanercept, Infliximab and Adalimumab inhibits germinal center formation upon SRBC immunization Pharmacological TNF neutralization with Infliximab results in loss of mycobacterial infection resistance in *M. tuberculosis* infection Pharmacological TNF inhibition with Etanercept or Infliximab reduces transplantable tumor growth of MCA205 fibrosarcoma and MDSC accumulation Pharmacological TNF neutralization with Infliximab protects mice from collagen antibody-induced arthritis Exacerbated disease and decreased Treg numbers in EAE	Pharmacological TNF inhibition decreases differentiation of CD11b^+^ cells into Ly6C^+^ monocytes and expression of genes encoding anti-apoptotic proteins *in vitro*	(82, 92–95)
hTNF x hTNFR2KI		Systemic (with the option of Cre-mediated hTNFR2 deletion in specific cell type)	Disease score and Treg numbers comparable to wild type mice in experimental autoimmune encephalomyelitis hTNFR2 deletion in FoxP3^+^ Tregs results in EAE exacerbation and malfunction of Tregs		(95)
hu/mTNFR1-k/i		Systemic	Treatment with TNFR1 antagonist protects cholinergic neurons against cell death and improves memory performance in a model of NMDA-induced neurodegeneration Treatment with TNFR1 antagonist inhibits EAE development and demyelination		(96, 97)
hu/mTNFR2-k/i		Systemic	Treatment with TNFR2 agonist protects cholinergic neurons against cell death and improves memory performance in a model of NMDA-induced neurodegeneration		(96)

### Mice With the Overexpression of Human TNF

The very first TNF humanized model was a transgenic mouse with the overexpression of TNF due to intentional dysregulation of TNF mRNA half-life (83). In general, overexpression of cytokines in animal models is a powerful tool to study molecular mechanisms associated with increased cytokine production (98). It is well established that dysregulated TNF production is detrimental in various autoimmune diseases including rheumatoid arthritis, psoriasis and IBD (99). Mice with human TNF overexpression (hTNF Tg mice with a high transgene copy number and dysregulated control) start to develop severe polyarthritis as early as 3-4 weeks after birth with similar characteristics observed in rheumatoid arthritis patients. Administration of antibodies to hTNF Tg mice led to the suppression of arthritis (83). Moreover, TNF overexpression in hTNF Tg mice led to increased incidence of spontaneous spinal disc herniation, which is involved in the development of acute radicular pain (84). These hTNF Tg mice displayed a decrease in hemoglobin associated with mild microcytic hypochromic anemia at the age of 9 weeks. Furthermore, TNF overexpression was associated with a decrease in the frequency of Sca-1^+^ progenitor cells and granulocytes with concurrent increase in the frequency of cells of both lymphoid and monocytic origin in the bone marrow (85). In summary, constitutive TNF overexpression is associated with the development of spontaneous autoimmune conditions. hTNF Tg mice served as an excellent model for associated disorders, such as progressive rheumatoid arthritis, although physiological relevance of using these mice in order to delineate the *in vivo* effects of systemic TNF inhibition on other functions was limited. To overcome the limitations of non-physiological levels of systemic TNF overproduction, other mouse models, such as tissue-specific, inducible and low copy number hTNF transgenic mice were developed.

Approaches to investigate tissue-restricted overexpression of human TNF in mice were also pioneered by G. Kollias group (88). For example, hTNF overexpression by T cells led to severe systemic effects, for example, CD2-hTNF Tg mice developed lethal progressive weight loss but no arthritis. hTNF overexpression by T cells also resulted in vascular thrombosis, tissue necrosis and lymphoid tissue abnormalities. Particularly, mice were characterized by reduced thymic cellularity and enlarged mesenteric lymph nodes that contained almost no lymphocytes. Overexpression of hTNF in astrocytes or neurons resulted in severe neurologic disease characterized by ataxia, seizures and relapsing hind limb paralysis (89). To delineate the contribution of soluble versus transmembrane TNF, mice that overexpress transmembrane human TNF in astrocytes (GFAP-tmTNF) or neurons (NFL-tmTNF) were engineered. Surprisingly, only astrocyte-specific overexpression of tmTNF was sufficient to trigger the development of neurologic disease (89). Thus, astrocytes appeared to be the source of pathogenic hTNF in a model of neuroinflammation.

The next step in the generation of hTNF transgenic mice was a low transgene copy number model characterized by low circulating levels of hTNF (86). In this case mice developed progressive arthritis at an older age, however, increased TNF production was also associated with reduced body weight, increased metabolic rate and restricted motor activity. This is similar to symptoms of rheumatoid cachexia in humans; therefore, these mice also represent a useful model to study conditions associated with elevated TNF production (100).

To overcome limitations of constitutive TNF overexpression, mice with reversible, doxycycline-inducible hTNF overexpression were generated. Two weeks after doxycycline administration, hTNF Tg mice developed psoriatic arthritis characterized by keratinocyte activation, joint and skin inflammation (87). Interestingly, signs of inflammation in this model were observed exclusively in the digits and, to a lesser extent, in the skin and ankles, unlike in mice with systemic TNF overexpression.

Another approach to generate mice with TNF overexpression was used by Liepinsh et al. (90). In this study mice with a large human genomic segment comprising hTNF and its two closest homologues, lymphotoxin α and β, were generated. Natural genomic context allowed hTNF/hLT genes to be expressed in response to physiological stimuli under the control of intrinsic regulatory elements. These mice demonstrated thymic atrophy and affected thymic T cell development with impaired thymocytes differentiation. Taken together, TNF humanized mouse models that partially mimic inflammatory conditions in patients with autoimmune disorders, such as rheumatoid arthritis and psoriasis, were generated and evaluated. Furthermore, to address the efficacy and possible side effects of TNF/TNFRs inhibitors in other disease models, humanized mice with regulated and cell type-specific hTNF expression were also established.

### Mice With Humanization of TNF and TNFR2

**Figure 2:**
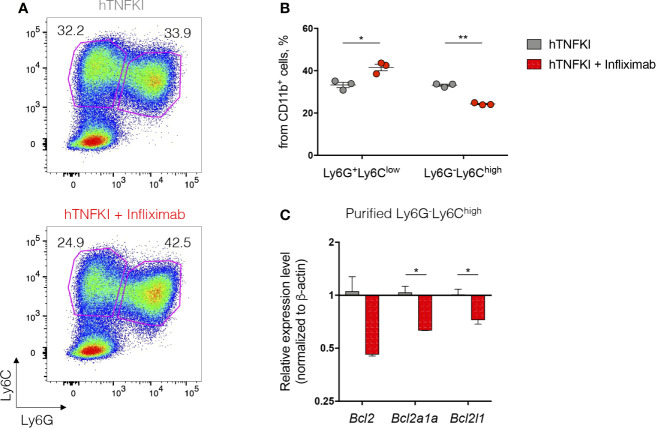
TNF inhibition affects immature myeloid cell development *in vitro*. Bone marrow cells were isolated from femurs of hTNFKI mice and cultured for 5 days in RPMI 1640 medium supplemented with L-Glutamine (2 mM), penicillin (100 U/ml), streptomycin (100 μg/ml), HEPES (10 mM), β-mercaptoethanol (50 μM), 10% FBS, GM-CSF (20 ng/ml) and IL-4 (10 ng/ml). Infliximab was added in the final concentration of 100 ng/ml. After 5 days in culture cells were stained with Fixable Viability Dye, CD11b (M1/70), Ly6C (HK1.4), Ly6G (RB6-8C5) and acquired with BD FACSCanto II flow cytometer. Data were analyzed using FlowJo software. **(A)** Representative FACS plots of Ly6G^+^Ly6C^low^ and Ly6G^-^Ly6C^high^ cells gated on VD^-^CD11b^+^ cells. **(B)** Frequencies of Ly6G^+^Ly6C^low^ and Ly6G^-^Ly6C^high^ cells gated on VD^-^CD11b^+^ cells. **(C)** Ly6G^-^Ly6C^high^ cells were purified using Myeloid-Derived Suppressor Cell Isolation Kit (Miltenyi Biotec) according to the manufacturer’s protocol. RNA was isolated from purified cells using TRIzol Reagent (Invitrogen) according to the manufacturer’s instructions. RNA (1 μg) was treated with DNase I and reverse transcribed to cDNA with M-MuLV reverse transcriptase (RevertAid first strand cDNA synthesis kit, Thermo Scientific). Real-time quantitative PCR was performed using qPCRmix-HS SYBR+LowROX (Evrogen) and the following primer set: *Actb*, Forward: CTCCTGAGCGCAAGTACTCTGTG, Reverse: TAAAACGCAGCTCAGTAACAGTCC, *Bcl2*, Forward: GAGTTCGGTGGGGTCATGTG, Reverse: TATAGTTCCACAAAGGCATCCCAG, *Bcl2a1a*, Forward: GGCAGAATGGAGGTTGGGAAG, Reverse: ATTCTCGTGGGAGCCAAGGT, *Bcl2l1*, Forward: AGAGAGGCAGGCGATGAGTT, Reverse: TCCACAAAAGTGTCCCAGCC. Reactions were run using the following program on the Applied Biosystems 7500: 95°C for 10 min, 40 cycles of 95°C for 15 sec, 61°C for 30 sec and 72°C for 20 sec. Each point in a diagram represents a single mouse; mean ± SEM. *P < 0,05; **P < 0,01. Two-tailed unpaired Student’s t-test was used.

Humanized TNF knock-in (hTNFKI) mice, in which case the mouse *Tnf* gene was substituted by its human ortholog, were generated using embryonic stem cell technology. They were used as a platform to study the effects of hTNF blockade in various disease models, including infectious, autoimmune, toxicity and transplantable tumor models (92, 93, 101, 102). Also, the efficacy of a novel myeloid-specific TNF inhibitor MYSTI in blocking hTNF and its effects in mouse models of LPS/D-galactosamine-induced hepatoxicity and collagen antibody-induced arthritis were demonstrated using these hTNFKI mice (93, 102). Additionally, these engineered mice allowed investigators to compare clinically available hTNF inhibitors such as Infliximab, Etanercept and Adalimumab (82). Furthermore, TNF ablation by pharmacological neutralization in hTNFKI mice led to the loss of the resistance to mycobacterial infection and to increased bacterial burden in the lungs (92). hTNF inhibition decreased tumor growth and MDSC accumulation in transplantable MCA 205 fibrosarcoma model, indicating a pro-tumorigenic function of TNF (94). Overall, hTNFKI mice are a useful tool to assess multiple effects of human TNF inhibition in various disease models, including adverse effects of TNF neutralization on hematopoietic compartment. To delineate the role of TNF inhibition with clinically approved blockers in myeloid cell differentiation, we isolated BM cells from hTNFKI mice, cultured them in the medium supplemented with GM-CSF and IL-4 with the addition of Infliximab and analyzed immature myeloid cell differentiation after 5 days of culturing (**Figure 2A**). We observed that TNF inhibition with Infliximab shifted differentiation of immature myeloid cells *in vitro*. Thus, TNF neutralization led to an increase in the frequency of Ly6G^+^Ly6C^low^ granulocytes and to a decrease in the frequency of Ly6G^-^Ly6C^high^ monocytes (**Figure 2B**). Since TNF is important for survival of monocytes (20), we hypothesized that decreased frequency of monocytes upon anti-TNF treatment was due to induction of apoptosis. To verify that, we analyzed expression of genes encoding anti-apoptotic proteins in purified Ly6G^-^Ly6C^high^ monocytes and found down-regulation of *Bcl2*, *Bcl2a1a* and *Bcl2l1* upon treatment with Infliximab (**Figure 2C**). Altogether, TNF blockade with Infliximab in BM cultures from hTNFKI mice inhibited the differentiation of immature myeloid cells into monocytes probably due to the induction of apoptosis.

Earlier biochemical studies indicated that hTNF can efficiently bind to TNFR1, but not to TNFR2 (103, 104). Indeed, inefficiency of hTNF interaction with murine TNFR2 led to disease exacerbation and decrease in Treg numbers in the periphery and CNS in a mouse model of multiple sclerosis (EAE), in which case TNFR2 signaling is protective (95). Therefore, it was desirable to generate a mouse with humanization of TNFR2 to provide efficient TNF-TNFR2 signaling. Mice were genetically designed to include two LoxP sites into human *TNFR2* locus, which allowed conditional Cre-mediated deletion of TNFR2 extracellular part in the desired cell type. These doubly humanized hTNF x hTNFR2KI mice showed EAE disease severity and Treg numbers comparable to wild-type mice, confirming the restoration of protective TNF/TNFR2 signaling. Cre-mediated genetic deletion of TNFR2 gene in Treg cells resulted in EAE exacerbation and malfunction of Treg cells. Intrinsic TNFR2 signaling was important for the maintenance of suppressive functions of Tregs by sustaining expression of Treg signature molecules, such as FoxP3, CD25, CTLA-4 and GITR (95). Further, hTNFR2 agonists applied to Treg cells from doubly humanized mice induced increased Treg cells proliferation (95).

Dong et al. also generated useful TNF receptor humanized mouse models, namely hTNFR1 knock-in and hTNFR2 knock-in mice, and demonstrated decreased neuroinflammation in response to TNFR1 antagonist ATROSAB or TNFR2 agonist EHD2-scTNFR2 (TNF hexamer oligomerized using the CH2 domain of IgE) in a model of NMDA-induced neurodegeneration (96). Additionally, ATROSAB administration inhibited development of EAE, decreased CNS infiltration and demyelination in hTNFR1KI mice (97). Yet another transgenic hTNFR1 mouse strain was used for studying the efficacy of VHH (antigen binding fragment of heavy chain only camelid antibody)-based nanobody against human TNFR1 (TNFR one silencer, TROS) in EAE, in which case a prophylactic administration of TROS resulted in disease amelioration (91).

In summary, there is a growing panel of useful humanized mouse models for evaluation of biologics that affect TNF/TNFR1/TNFR2 systems, including their effects on hematopoiesis. Furthermore, restoration of affected signaling by humanization of both TNF and its receptor, TNFR2, makes it possible to comparatively evaluate not only anti-TNF drugs, but also hTNFR2 agonists and antagonists. Taken together, humanized mouse models will allow investigators to study efficacy of various TNF/TNFRs-targeting biologics and assess possible side effects on other systems for further clinical translation.

## Concluding Remarks

For many years TNF was mainly considered a proinflammatory cytokine with its role in host defense, but also with detrimental effects on autoimmunity. However, basic studies on TNF biology, as well as reported side effects in patients receiving anti-TNF therapy, highlighted its homeostatic functions in many physiological processes, including hematopoiesis. Regulation of TNF/TNFRs expression in various tissues modulates the cross-talk between immune and non-immune cells, which subsequently determines the outcome of TNF action.

Future investigation of pathological versus regulatory functions of TNF and deciphering its systemic and local effects in tissues may help to improve current therapeutic approaches. Therefore, mouse models with humanized TNF/TNFRs system represent a powerful tool to study side effects of anti-TNF therapy on hematopoiesis.

## Author Contributions

VG, KA, MD, and SAN designed research. VG and KA. performed research and analyzed data. VG, KA, AD, TY, MSD, and SN discussed the concept and wrote the manuscript. All authors contributed to the article and approved the submitted version.

## Funding

Reverse genetics studies were supported by the grant 075-15-2019-1660 from the Ministry of Science and Higher Education of the Russian Federation, myelopoiesis studies were supported by RFBR grant 19-34-51030.

## Conflict of Interest

The authors declare that the research was conducted in the absence of any commercial or financial relationships that could be construed as a potential conflict of interest.
